# Curvelet Decomposition-Based Tri-Branch Coupling Network for Hyperspectral Unsound Maize Seeds Identification

**DOI:** 10.3390/foods15111859

**Published:** 2026-05-24

**Authors:** Kuibin Zhao, Lei Lu, Pengtao Lv, Hongyi Ge

**Affiliations:** 1 College of Information Science and Engineering, Henan University of Technology, Zhengzhou 450001, China; kbzhao@stu.haut.edu.cn (K.Z.); pengtaolv@163.com (P.L.); gehongyi2004@163.com (H.G.); 2 Institute for Complexity Science, Henan University of Technology, Zhengzhou 450001, China; 3 Key Laboratory of Grain Information Processing and Control, Henan University of Technology, Ministry of Education, Zhengzhou 450001, China

**Keywords:** curvelet transform, information fusion, Mamba feature, unsound seeds, maize identification

## Abstract

The rapid and nondestructive classification of maize kernels is of great significance for seed screening and quality evaluation. Existing hyperspectral image classification methods based on the Mamba architecture can effectively represent spectral and spatial features; however, they still face limitations in time–frequency analysis and multimodal feature fusion. In addition, traditional approaches often rely heavily on spectral preprocessing, which may introduce additional errors and compromise the model’s robustness and generalization ability. To address these challenges, this paper proposes a novel cross-modal classification framework named CD-TriMamba, which jointly leverages hyperspectral data and visible-light images for comprehensive feature extraction and deep fusion. Specifically, an innovative feature extraction module is designed, consisting of a Spectral Curvelet Convolution (SCC) module for hyperspectral data and a Curvelet-Decomposed Convolution (CDC) module for spatial modeling. A feature rearrangement mechanism is further introduced to mine critical information from both spectral and spatial modalities. Finally, a ConvNeXt-guided tri-branch cross-fusion structure (TriMamba) is constructed to achieve deep collaboration and efficient integration between spectral and spatial features. Experimental results demonstrate that the proposed model achieves outstanding performance in seed classification, with an accuracy (Acc) of 99.2% and a Kappa value of 99.1%. These results strongly confirm the effectiveness and broad application potential of cross-modal feature fusion in maize kernel classification.

## 1. Introduction

Although existing studies have achieved considerable progress in maize kernel classification, current methods still face challenges in distinguishing subtle defect differences under complex imaging conditions. In addition, the integration of frequency-domain feature decomposition and long-range dependency modeling in hyperspectral analysis remains insufficiently explored, limiting the representation capability and robustness of existing approaches [1]. Against this background, the classification and identification of maize kernels have increasingly become a research hotspot. Its significance is reflected in several aspects: on the one hand, it is crucial for ensuring the accuracy and reliability of grain quality inspection and storage management [2]; on the other hand, it provides direct support for seed screening, breeding improvement, and quality evaluation [3,4]. With the rapid development of artificial intelligence and machine vision technologies, maize kernel classification and recognition have shown broad application prospects [5], not only in grain quality inspection and storage but also in automated grading, seed processing, and intelligent storage, effectively improving efficiency and reducing labor costs [6]. Traditional seed identification methods mainly rely on physical, chemical, and physiological approaches. However, these methods have certain limitations: on the one hand, they often require destructive sampling, preventing subsequent planting or utilization; on the other hand, the procedures are time-consuming and labor-intensive, making them unsuitable for large-scale, rapid screening in modern agriculture. Consequently, the development of efficient and nondestructive detection methods has become a major focus of recent research.

In recent years, nondestructive detection technologies have been widely applied in seed variety identification. Among them, machine vision methods have been the earliest adopted techniques due to their low cost, high speed, and ease of implementation [2]. Research based on image processing and deep learning has made significant progress, such as classifying maize kernels using texture and morphological features [7], improving recognition accuracy with convolutional neural networks [8], and representing kernel appearance differences through multi-view imaging. However, machine vision primarily relies on external appearance features, which are often insensitive to internal quality differences and susceptible to variations in illumination, viewing angle, and surface defects, thereby limiting robustness and generalization.

To address these limitations, near-infrared (NIR) spectroscopy has been widely applied in seed detection. NIR captures chemical composition characteristics of kernels based on molecular vibrations, overcoming the limitations of methods that rely solely on appearance [9]. Previous studies have demonstrated that NIR can rapidly predict kernel moisture, protein content, and variety [10]. Nevertheless, a major drawback of NIR is its lack of spatial resolution, making it challenging to handle kernel mixing and positional uncertainty in complex backgrounds. Hyperspectral imaging (HSI) builds on this by combining spectral information with spatial structure, enabling analysis of both internal composition and image-level spatial features. Consequently, HSI exhibits significant advantages in seed quality assessment and classification [11]. However, HSI also faces challenges such as high-dimensional data redundancy, high computational complexity, and low modeling efficiency, which limit its scalability in large-scale applications [12].

Driven by advances in hyperspectral imaging, the integration of HSI and deep learning has become an important research direction for improving seed identification accuracy and robustness. For instance, CNN-LSTM-based methods combine spatial feature extraction and spectral dependency modeling to achieve high-accuracy maize variety classification [13]. In sweet maize kernel vigor detection, various network architectures, including 1D-CNN, LSTM, and CNN-LSTM, were compared, and model performance was further enhanced using intelligent optimization algorithms, demonstrating the advantages of deep learning in complex kernel state recognition [14]. Meanwhile, hybrid convolution–Transformer architectures have also been introduced for hyperspectral modeling to balance local texture extraction with global dependency modeling, although at the cost of high computational complexity [15,16]. Recently, the Mamba structure within state-space models (SSM) has been applied to hyperspectral image classification, showing potential in modeling long-range dependencies and improving inference efficiency [17,18]. However, a single Mamba model still struggles to capture multi-scale textures and local details; in contrast, wavelet-based multi-scale approaches can effectively reduce data redundancy but often compromise structural information, making it challenging to fully represent complex seed appearance and internal composition.

To address these challenges, we introduce a spectral–spatial framework that integrates curvelet decomposition with the Mamba architecture for hyperspectral maize kernel classification. Curvelet decomposition enhances multi-scale directional and geometric feature representation, while the Mamba structure effectively captures long-range spectral–spatial dependencies and global contextual information. Different from CNN-based methods that primarily focus on local feature extraction and Transformer-based methods that rely heavily on global self-attention mechanisms, the proposed approach achieves a more balanced modeling of both local fine-grained structures and global contextual dependencies.

Our main contributions are as follows:

- A spectral–spatial collaborative convolution (SCC) module combining Curvelet decomposition and convolution operations was designed to enhance multi-scale spectral–spatial feature representation, thereby improving the perception and extraction capability for subtle defect information.
- A cross-domain dependency coupling (CDC) module integrating separable spectral–spatial convolution with the Mamba architecture was proposed to effectively model long-range global dependencies while reducing computational complexity and redundant information.
- A novel CD-TriMamba network with parallel spectral–spatial branches and cross-domain feature fusion mechanisms was developed to achieve efficient multimodal feature interaction and comprehensive representation learning, thereby enhancing the discriminative capability for maize kernel classification tasks.
- Extensive comparative and ablation experiments were conducted on maize hyperspectral datasets. The experimental results demonstrate that the proposed method consistently outperforms existing state-of-the-art methods in terms of classification accuracy, robustness, and generalization capability.

The structure of this paper is organized as follows: Section 2 reviews the relevant background work; Section 3 provides a detailed description of the proposed CD-TriMamba model architecture and methodology; Section 4 presents the experimental design, results analysis, and discussion; finally, Section 5 concludes the paper and outlines directions for future research.

## 2. Related Work

### 2.1. Curvelet Decomposition

In recent years, machine vision–based nondestructive detection methods have achieved remarkable progress in seed variety identification by integrating texture, morphological, and multi-view appearance features with deep learning techniques. However, these methods primarily rely on external appearance information and are often insensitive to subtle internal quality variations. Moreover, they are highly susceptible to changes in illumination, viewing angle, and surface defects, which limits their robustness and generalization capability in complex agricultural scenarios.

To address these limitations, frequency-domain analysis methods have attracted increasing attention in hyperspectral and visual representation learning. Among them, the Curvelet transform has demonstrated strong capability in multi-scale and multi-directional signal analysis, particularly in representing geometric structures and curved edge information with high precision. This property enables effective extraction of directional and spectral–spatial frequency-domain features [19]. In recent years, the integration of curvelets with deep learning has demonstrated great potential in various visual tasks [20,21]. In these studies, curvelets not only compensate for the limitations of traditional transforms in multi-resolution geometric analysis but can also be seamlessly embedded into deep network architectures, significantly enhancing the model’s capability in extracting complex structural features.

In the field of hyperspectral imaging (HSI), curvelet transform has been widely employed for extracting multi-scale and multi-directional features to enhance spectral and spatial representations. Ma et al. [22] proposed a curvelet framework that effectively captures curved edge structures in images while maintaining sparsity. Qiao et al. [23] applied curvelet transform to image denoising and demonstrated its superiority in edge preservation and detail recovery. In subsequent studies, curvelet decomposition features have gradually been integrated into deep networks to complement convolutional features, thereby exhibiting stronger discriminative capability in hyperspectral feature extraction and classification tasks [24].

**Figure 1:**
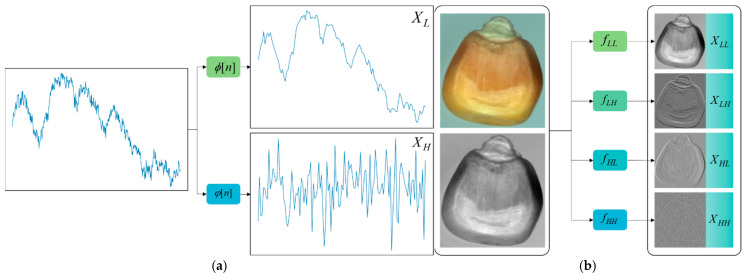
(**a**) Schematic of 1-D db2 wavelet decomposition. (**b**) Schematic of 2-D db2 wavelet decomposition.

For a one-dimensional signal F[n], applying db2 wavelet decomposition yields low-frequency signal $X_{L}$ and high-frequency signal $X_{H}$ through convolutions with low-pass and high-pass filters, as illustrated in Figure 1. The low-frequency component $X_{L}$ typically represents the overall trend or smooth portion of the signal and is extracted via the convolution of F[n] with ϕ[n]. The high-frequency component $X_{H}$ emphasizes signal details and instantaneous variations and is extracted via the convolution of F[n] with φ[n]. The process can be expressed mathematically as follows:

(1) $$X_{L}[k]=F[n{]}^{*}\phi [n]=\sum_{n}F[n]\cdot \phi [2k-1]$$

(2) $$X_{H}[k]=F[n{]}^{*}\varphi [n]=\sum_{n}F[n]\cdot \varphi [2k-1]$$

In Equations (1) and (2), ϕ[n] denotes the low-frequency convolution kernel, which preserves the low-frequency information of the signal, while φ[n] denotes the high-frequency convolution kernel, which captures the high-frequency components. By decomposing the spectral signal into complementary low- and high-frequency parts, subtle variations in the spectrum can be characterized, revealing complex features across different frequency bands. For two-dimensional maize images, the basic principle of db2 wavelet decomposition is similar to that of one-dimensional wavelet decomposition, but it requires applying low-pass and high-pass filtering along both horizontal and vertical directions. Specifically, the process begins by performing low-pass and high-pass filtering followed by down-sampling in the horizontal direction, yielding low-frequency (L) and high-frequency (H) sub-bands. Subsequently, these two sub-bands are filtered and down-sampled along the vertical direction, resulting in four sub-bands. This decomposition enables effective multi-scale and multi-directional representation of maize images, capturing both overall contours and local details [25].

To process hyperspectral data, researchers have further introduced 2-D/3-D discrete wavelet transforms (DWT) [26]. For example, Xu et al. [27] employed 3-D DWT to simultaneously extract joint spectral and spatial frequency features, using the resulting block-level wavelet features as overall training parameters. Another study [28] applied multi-level wavelet decomposition to each image block to obtain multi-scale spectral–spatial features, enabling a more efficient feature model within a Transformer network. In 2-D wavelet decomposition, low-pass and high-pass filters are applied along the horizontal and vertical directions to perform the decomposition, as shown in Equation (3):

(3) $$\left[\begin{array}{cc} X_{LL} & X_{LH}\\ X_{HL} & X_{HH} \end{array}\right]=\left[\begin{array}{cc} X^{*}f_{LL} & X^{*}f_{LH}\\ X^{*}f_{HL} & X^{*}f_{HH} \end{array}\right]$$

Using four convolution kernels, the maize image X is decomposed into four sub-images: one low-frequency component $X_{LL}$ and three high-frequency components $X_{LH}$, $X_{HL}$, and $X_{HH}$, corresponding to approximation information, horizontal details, vertical details, and diagonal details, respectively. The corresponding convolution kernels are as follows: $f_{\mathrm{LL}}=\frac{1}{2}\left[\begin{array}{cc} 1 & 1\\ 1 & 1 \end{array}\right]$, $f_{\mathrm{LH}}=\frac{1}{2}\left[\begin{array}{cc} 1 & -1\\ 1 & -1 \end{array}\right]$, $f_{\mathrm{HL}}=\frac{1}{2}\left[\begin{array}{cc} 1 & 1\\ -1 & -1 \end{array}\right]$, and $f_{\mathrm{HH}}=\frac{1}{2}\left[\begin{array}{cc} 1 & -1\\ -1 & 1 \end{array}\right]$.

### 2.2. Mamba Network

The traditional state-space model (SSM) originates from control theory. SSM describes the current input sequence $x(t)\in R^{N}$ through a hidden state h(t)∈R, and its continuous-time system can be expressed as follows:

(4) $$h^{\prime }(t)=Ah(t)+Bx(t),\;y(t)=Ch(t)+Dx(t)$$

Here, the coefficient matrix $A\in R^{N\times N}$ represents the state transition term, $B\in R^{N\times 1}$ represents the control input term, $C\in R^{1\times N}$ represents the observation output term, and D∈R denotes the feedback term. In deep learning, these coefficients are typically treated as learnable parameters after discretization. For continuous-time systems, assuming the input remains constant over the time interval Δt, the zero-order hold (ZOH) method can be used to discretize the continuous system. The discretized system is expressed as follows:

(5) $$h_{k}=\bar{A}h_{k-1}+\bar{B}x_{k},y_{k}=Ch_{k}+Dx_{k}$$

In the above system, $\bar{A}=e^{A\Delta t}$ and $\bar{B}=\left(\int_{0}^{\Delta t}e^{A\tau }d\tau \right)B$, the parameters of traditional SSMs, are typically time-invariant, which limits their capability to handle sequences with complex dynamic variations. To address this issue, the selective S6 model was proposed [29], the core idea of which is to design key parameters as functions of the input sequence, enabling the model to dynamically adapt its structure according to the data.

Although previous studies have independently demonstrated the effectiveness of frequency-domain transformations for multi-scale spectral–spatial feature extraction and the superiority of Mamba networks for long-range dependency modeling, their synergistic integration in natural hyperspectral image (HSI) analysis remains largely unexplored. To bridge this gap, this study proposes a novel dual-domain Mamba framework specifically designed for imperfect maize kernel recognition. Unlike conventional convolution-based or Transformer-based models, the proposed method innovatively embeds wavelet-derived subbands, which are effective in capturing complex local geometric details and abrupt spectral variations, into the selective state-space mechanism of Mamba for dynamically modeling global contextual dependencies across continuous spectral bands. This unique fusion of frequency-domain decomposition and state-space sequential modeling not only significantly enhances the discriminative representation of subtle kernel defects but also provides an efficient and comprehensive solution for hyperspectral agricultural inspection tasks.

## 3. Methods

This section provides a detailed description of the proposed CD-TriMamba network. First, we present an overview of the overall framework and workflow of the model; subsequently, we discuss the design motivations and specific implementations of several key components.

**Figure 2:**
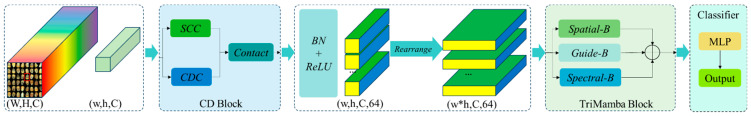
Schematic of the proposed CD-TriMamba network. “CDC” is the curvelet decomposition convolution module used for extracting spatial features. “SCC” is the spectral curvelet convolution module used for extracting spectral features. CDC and SCC both have a size of w*h*32. “CD block” concatenates the outputs of CDC and SCC, in CD block, “contact” refers to the concatenation operation along the channel dimension, thus generating the final feature map of size w*h*64. “TirMamba block” performs spectral-spatial feature fusion, “MLP” is a multilayer perceptron layer used for final classification.

Figure 2 illustrates the overall architecture of the proposed CD-TriMamba model. At the input stage, the original image is divided into patches of size w×h×C to fully exploit the spatial information within local pixel neighborhoods. These patches are then fed into the CD module, which consists of two parallel submodules: the CDC and SCC modules. The CDC module integrates convolution with a 2-D curvelet transform to capture spatial structural features in the frequency domain, while the SCC module combines convolution with a 1-D curvelet transform to characterize subtle variations along the spectral dimension, enhancing the model’s ability to discriminate between different material categories. Outputs from the CDC and SCC modules are concatenated to form a more refined feature representation. Compared with 3-D wavelet transforms, this approach reduces computational complexity by separately employing 1-D and 2-D curvelet decompositions: the 1-D curvelet focuses on spectral feature extraction, whereas the 2-D curvelet is dedicated to spatial feature modeling, enabling more targeted feature representation.

### 3.1. Overall Framework of CD-TriMamba

Within the TriMamba block, the fused features are first rearranged and then input into a three-branch structure based on the Mamba architecture: the spatial Mamba branch and the spectral Mamba branch. The former emphasizes spatial dependency modeling, while the latter captures long-range correlations along the spectral dimension. The introduction of residual connections ensures feature integrity during propagation. At the output stage, spectral features, spatial features, and the original input features are further fused via residual mechanisms, and the resulting multi-level features are passed to a multilayer perceptron (MLP) to perform the classification task.

**Figure 3:**
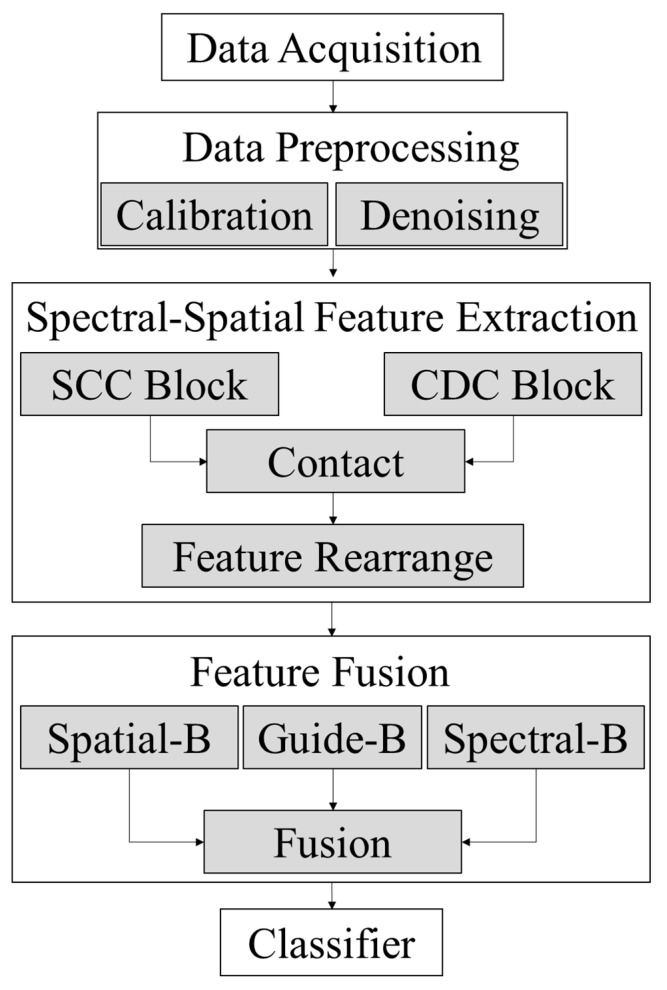
The overall workflow of the CD-TriMamba Network.

To provide a comprehensive and intuitive overview of the aforementioned processes, the complete operational flowchart of the proposed framework is illustrated in Figure 3. This diagram systematically delineates the entire pipeline, from initial hyperspectral data acquisition and preprocessing to the final classification, explicitly highlighting the structural integration of the dual-domain Mamba modeling and cross-domain feature fusion.

Additionally, to balance computational efficiency and the nonlinear characteristics of hyperspectral data, the model incorporates Batch Normalization and ReLU activation functions in several key layers, further enhancing training stability and feature representation capability.

### 3.2. Curvelet Decomposition Block

As early as 1999, E.J. Candes and Donoho proposed the first-generation curvelet transform framework, which was developed based on the Ridgelet theory [19]. The curvelet transform aims to achieve superior approximation performance and outperforms traditional wavelet transforms [21]. It is particularly effective in representing information with curved shapes or rich edge features. Unlike wavelet transforms, the continuous curvelet transform retains translation and scale parameters while introducing a directional parameter, thereby enabling directional discrimination [30]. Specifically, a sparse representation of the signal is achieved via the inner product between the signal and the functions:

(6) $$C_{j,l,k}=<f,\varphi_{j,l,k}>$$

Here, j,l,k correspond to scale, orientation, and position, respectively. The 2-D curvelet transform decomposes an image into a series of non-overlapping scales and analyzes each scale using local Ridgelet transforms. To accommodate digital processing, the continuous curvelet transform is discretized, taking a Cartesian grid as input and producing a set of coefficients as output. Specifically, let $f[t_{1},t_{2}]$ denote the input signal, with $0<t_{1}<n$ and $0<t_{2}<n$ representing the spatial coordinates of the input signal. The curvelet transform coefficients are then given by:

(7) $$C_{Discrete}(j,l,k)=\sum_{t=0,t_{1}=0}^{n}f[t_{1},t_{2}]\varphi_{D}(j,l,k)[t_{1},t_{2}]$$

In the equation, $\varphi_{D}(j,l,k)$ denotes the basis function of the discrete curvelet transform; its corresponding representation in the frequency domain can be expressed using a defined local window function as:

(8) $${\hat{U}}_{j}(\omega )={\hat{W}}_{j}(\omega )\cdot V_{j}(\omega )$$

In the equation, ω denotes the frequency-domain parameter, $W_{j}=\sqrt{\varphi_{j}^{2}+\omega +\varphi_{j}^{2}(\omega )}$ the radial function, $V_{j}=V(2^{\left|j/2\right|}\omega_{1}/\omega_{2})$ the angular function, and φ represents a one-dimensional inner product, with:

(9) $$\varphi^{j}(\omega_{1},\omega_{2})=\varphi (2^{-j}\cdot \omega_{1})\cdot \varphi (2^{-j}\cdot \omega_{2})$$

The transformation is performed in polar coordinates:

(10) $${\hat{V}}_{j,q}(\omega )={\hat{W}}_{j}(\omega )\cdot V_{j}(S_{\theta q}\omega )$$

In the equation $S_{\theta q}=\left[\begin{array}{cc} 1 & 0\\ -\tan\theta q & 1 \end{array}\right]$, θ denotes the frequency-domain polar coordinates, and θq represents the angular sequence. The discrete curvelet function can thus be defined as:

(11) $$\varphi_{j,l,k}(X)=\varphi_{j}\left[\left(S_{\theta q}^{T}(X-S_{\theta q}^{T}\cdot b)\right)\right]$$

In the equation, $b=(2^{-l}k_{1}x,2^{-l}k_{2}x)$ and x denote position parameters, while $k_{1}$ and $k_{2}$ represent translation parameters.

**Figure 4:**
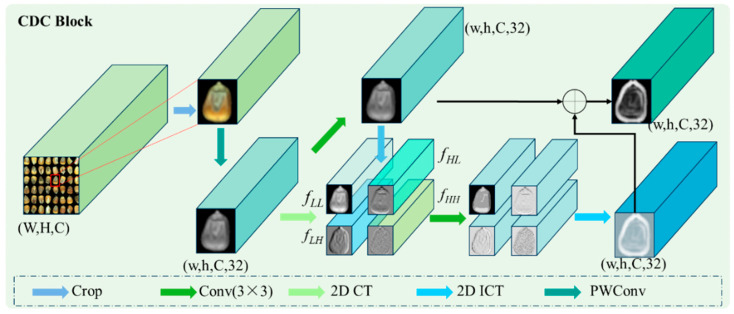
Spatial feature extraction of the CDC Block.

The curvelet transform, as a powerful multi-scale decomposition tool, is particularly suitable for image data with curved and edge structures. Based on the curvelet transform theory, as illustrated in Figure 4, the CDC integrates pointwise convolution to achieve efficient computation of the 2-D curvelet transform, effectively extracting spatial frequency-domain information while maintaining high computational efficiency.

Specifically, for the input image $X_{input}\in R^{W\times H\times C}$, a pointwise convolution layer is first applied to reduce its spatial dimensions, generating an optimized feature map. Subsequently, the db2 wavelet transform is applied to the down-sampled feature map to achieve multi-scale decomposition across different frequency bands, producing four sub-band components: LL, LH, HL, and HH, which correspond to low- and high-frequency information. Each sub-band component is then further processed through convolution layers with 3×3 kernels $W_{1}$, effectively extracting local fine-grained features. After this processing, the feature maps are restored to their original spatial dimensions via a 2-D inverse wavelet transform, resulting in the complete feature map. The mathematical derivation is as follows:

(12) $$X_{2d-ct}={\mathrm{ICT}}_{2d}\left(\mathrm{Conv}\left(W_{1},\left[\begin{array}{cc} X_{\mathrm{LL}} & X_{\mathrm{LH}}\\ X_{\mathrm{HL}} & X_{\mathrm{HH}} \end{array}\right]\right)\right)$$

To enhance the model’s performance, the output of the CDC module employs a custom residual connection strategy. Specifically, the down-sampled feature map is first convolved with another set of convolution kernels $W_{2}$, and then fused with the output of the 2-D Curvelet Transform (2D-CT), resulting in the final output of the CDC module. The fusion process is mathematically expressed as follows:

(13) $$X_{Conv2d}=\mathrm{Conv}\left(W_{2},\mathrm{PWConv}\left(X_{Input}\right)\right)$$

(14) $$X_{CDC}=X_{2d-ct}+X_{Conv2d}$$

In this manner, the curvelet transform not only captures fine-grained details within the image but also effectively reduces computational complexity and achieves precise decomposition in the spatial frequency domain, ultimately enhancing the efficiency and accuracy of image processing tasks.

The CDC module effectively captures key image features, such as shapes and contours, through the application of the 2-D curvelet transform. Its multi-scale decomposition capability enables the model to extract fine-grained information across different frequency levels, enhancing sensitivity to details. Furthermore, the module strengthens the extraction of local features, effectively capturing fine details while ensuring precise recognition of complex image structures, as illustrated in Figure 4.

### 3.3. Spectral Curvelet Convolution

**Figure 5:**
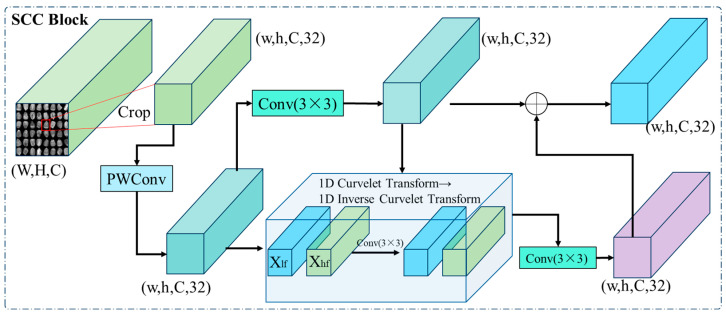
Spectral feature extraction of the SCC Block.

Unlike conventional spatial image processing, hyperspectral images contain rich spectral information, with each pixel exhibiting responses across multiple bands that together form its unique spectral signature. To further enhance spectral feature extraction in hyperspectral imaging (HSI), we introduce the SCC module. This module combines one-dimensional curvelet decomposition with convolution operations, enabling effective multi-scale capture of spectral features. The structure of the module is illustrated in Figure 5.

For spectral feature extraction, we propose the Spectral Curvelet Convolution (SCC) module. First, the input image $X_{input}\in R^{W\times H\times C}$ undergoes rotated convolution along the spectral dimension to reduce redundant spectral information while preserving essential frequency details. Based on extensive experiments, the initial number of convolution channels is set to 32 to achieve an optimal balance between model complexity and performance. Subsequently, the SWC module applies a one-dimensional discrete curvelet transform along the spectral dimension, decomposing the reduced spectral feature map into a low-frequency component $X_{lf}$ and a high-frequency component $X_{hf}$. The low-frequency component captures the overall trend of the spectral curve, whereas the high-frequency component encodes fine-grained perturbations and local variations, enabling multi-scale modeling of both coarse and fine spectral features. After decomposition, the low- and high-frequency components are separately convolved using the $W_{1}\in R^{1\times 1\times 3}$ convolutional kernel to further extract discriminative features at different frequency scales. This convolutional kernel effectively models correlations between adjacent spectral bands while maintaining the model’s expressive capacity with reduced computational cost and parameter size. Finally, the features are reintegrated into the original spectral dimension via the one-dimensional inverse curvelet transform (1-D IDCT), achieving multi-scale spectral feature fusion. In this way, the SCC module not only emphasizes the global spectral trend but also enhances sensitivity to local details, thereby improving feature representation in hyperspectral imaging tasks. The mathematical formulation is as follows:

(15) $$X_{1d-ct}={\mathrm{ICT}}_{1d}\left(\mathrm{Conv}\left(W_{1},\left[X_{lf},X_{hf}\right]\right)\right)$$

On this basis, the SCC module further employs a residual fusion strategy, summing the one-dimensional curvelet-transformed feature map $X_{1d-ct}$ with the convolutional output feature map $X_{Conv1d}$ to produce the final output of the module:

(16) $$X_{Conv1d}=\mathrm{Conv}\left(W_{2},\mathrm{PWConv}\left(X_{Input}\right)\right)$$

(17) $$X_{SCC}=X_{1d-ct}+X_{Conv1d}$$

The SCC module fully exploits the advantages of the one-dimensional Curvelet Transform (1-D CT) in spectral frequency decomposition, significantly enhancing the model’s sensitivity to complete spectral information. While maintaining low computational complexity, the SCC module captures spectral features more comprehensively, thereby improving classification performance. The frequency-domain features extracted via curvelet decomposition provide rich multi-scale contextual information for subsequent Mamba blocks. In turn, the SSM mechanism within the Mamba blocks dynamically adapts its parameters to enhance selective perception of frequency-domain features, adjusting sensitivity to different spectral frequencies based on the input data to more accurately extract critical spectral information.

### 3.4. TriMamba Block

**Figure 6:**
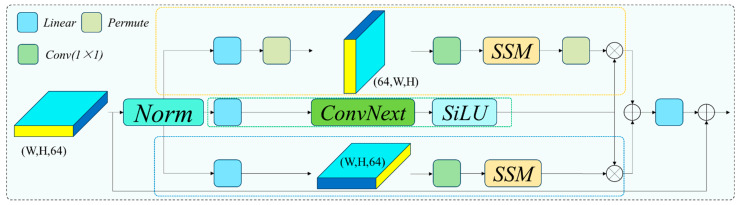
The TriMamba block has three parallel branches: spatial branch and spectral branch.

The overall architecture of the TriMamba block is illustrated in Figure 6 and consists of three parallel paths: the spectral Mamba branch, the spatial Mamba branch, and the guiding branch. The input feature map is first normalized and then fed into the three branches to fully capture spectral, spatial, and global information, which are subsequently modeled via the SSM mechanism. To improve the efficiency of Mamba in modeling long-range dependencies, the features undergo structural preprocessing before entering the SSM. First, the extracted 3-D feature block $X\in R^{W\times H\times C}$ is scanned along one dimension and systematically unfolded into a 2-D representation $X\in R^{N\times D}$.

This process reduces the data dimensionality while effectively preserving key spectral and spatial information. Compared with directly processing high-dimensional tensors, this transformation compresses the 3-D features into a unified token sequence, providing a more structured input for the subsequent Mamba encoder and allowing the state-space model to fully leverage its capabilities in long-range dependency modeling and global context capture. Unlike traditional spectral–spatial models that rely on fixed scanning strategies, the TriMamba module adopts a more flexible representation. During the subsequent Mamba encoding stage, dynamic axial permutation is introduced, enabling the model to adaptively select the optimal scanning dimension and modeling path based on the input data. This mechanism not only avoids information loss associated with fixed strategies but also significantly enhances Mamba’s representational power and robustness in joint spectral–spatial modeling.

In the spectral Mamba branch, the normalized feature sequence first passes through a linear layer followed by a 1 × 1 convolution. The linear layer performs feature standardization and recalibration to enhance numerical stability, while the 1 × 1 convolution enables feature compression and dimensional transformation through inter-channel interactions, thereby improving representation capacity. This design not only effectively integrates local correlations along the spectral dimension but also provides a more compact and discriminative feature representation for the subsequent selective scanning in the modeling stage. The computation is given as follows:

(18) $$X_{Spe-B}={\mathrm{Conv}}_{1\times 1}\left(\mathrm{Linear}\left(\mathrm{Norn}\left(X_{Input}\right)\right)\right)$$

Subsequently, the processed feature sequence $X_{Spe-B}\in R^{N\times D}$ is fed into the state-space model for representation learning. In the spectral Mamba branch, the SSM employs a parameterized recursive state update mechanism to effectively capture the dynamic dependencies among features along the spectral dimension, which can be formally expressed as:

(19) $$Y_{Spe}=\mathrm{SSM}\left(A_{1},B_{1},C_{1},X_{Spe-B}\right)$$

The processing flow of the spatial Mamba branch is similar to that of the spectral branch, with one key distinction: prior to entering the SSM, the feature sequence is first transposed to rearrange the data from the spectral dimension to the spatial dimension. This enables the SSM to capture long-range dependencies among features at different spatial locations, facilitating cross-region contextual modeling. Through this process, the model’s perception of global spatial information is significantly enhanced, providing more expressive spatial representations for subsequent discriminative tasks, which can be computed as follows:

(20) $$X_{Spa-B}={\mathrm{Conv}}_{1\times 1}\left(\mathrm{Linear}\left(\mathrm{Norn}{\left(X_{Input}\right)}^{T}\right)\right)$$

(21) $$Y_{Spa}=\mathrm{SSM}\left(A_{2},B_{2},C_{2},X_{Spa-B}\right)$$

In the guiding branch, a *ConvNeXt* classifier is employed, and the SSM outputs are modulated using the *SiLU* activation function, computed as follows:

(22) $$Y_{Tuner}=\mathrm{SiLU}\left(\mathrm{ConvNext}\left(\mathrm{Linear}\left(\mathrm{Norn}\left(X_{Input}\right)\right)\right)\right)$$

(23) $$Y=\mathrm{Linear}\left(Y_{Spe}⊙Y_{Tuner}+Y_{Spe}⊙Y_{Tuner}\right)+X_{Input}$$

The modulation introduced by the guiding branch enhances the nonlinear relationships within the feature representations, playing a crucial role in subsequent fusion. The activated spectral and spatial features are then effectively fused via element-wise multiplication, ensuring comprehensive integration of multi-dimensional information. Through linear projection and residual connections, the fused representation is further combined with the original input features. Specifically, the residual connections not only systematically preserve the original information but also allow the fused representation to complement the input features, maximizing the interaction across the two-dimensional feature space. This deep fusion mechanism not only strengthens the model’s ability to understand and leverage complex feature relationships but also significantly improves classification performance. Leveraging this architecture, the TriMamba block achieves efficient and comprehensive joint modeling of spectral and spatial information under the core guidance of the guiding branch.

Within the TriMamba block, Linear modules are strategically positioned at different locations to perform their specific functions, collaboratively enabling parallel modeling of spectral and spatial feature sequences. This meticulous arrangement not only enhances the model’s capability to capture long-range dependencies but also maintains high computational efficiency, resulting in significant improvements in classification accuracy and model robustness for hyperspectral image classification tasks.

### 3.5. Loss Functions

To supervise the entire training process, the model employs the cross-entropy loss as the optimization objective. This loss function effectively guides the network to learn discriminative features between correct and incorrect hand posture images by quantifying the discrepancy between predicted outputs and true labels, thereby enhancing overall classification performance. The cross-entropy loss is computed as follows:

(24) $$L_{\mathrm{cls}}=y\log\hat{y}+(1-y)\log(1-\hat{y})$$

Here, y denotes the ground-truth label of the hand posture image, and $\hat{y}$ represents the label predicted by our method. When the image is misclassified, the label is set to y=0; when the image is correctly classified, the label is set to y=1.

To fully exploit the learning potential of each feature extraction branch, we designed a multi-branch loss strategy, assigning an independent loss function to each branch. Specifically, the spectral feature extraction branch corresponds to the loss $L_{Spe}$, the spatial image branch corresponds to $L_{Spa}$, and the guide branch corresponds to $L_{Guide}$. Each loss function is specially designed for the feature dimensions of its respective branch, enabling targeted optimization of different types of features and maximizing the representational power of each branch. The loss functions for each branch are defined as follows:

(25) $$L_{Spe}=y_{Spe}\log{\hat{y}}_{Spe}+(1-y_{Spe})\log(1-{\hat{y}}_{Spe})$$

(26) $$L_{Spa}=y_{Spa}\log{\hat{y}}_{Spa}+(1-y_{Spa})\log(1-{\hat{y}}_{Spa})$$

(27) $$L_{\mathrm{Guide}}=y_{\mathrm{Guide}}\log{\hat{y}}_{\mathrm{Guide}}+(1-y_{\mathrm{Guide}})\log(1-{\hat{y}}_{\mathrm{Guide}})$$

(28) $$L=L_{cls}+\lambda_{1}L_{Spe}+\lambda_{2}L_{Spa}+\lambda_{3}L_{\mathrm{Guide}}$$

Here, $\lambda_{1}$, $\lambda_{2}$, and $\lambda_{3}$ are hyperparameters used to balance the contribution of each branch’s loss. By default, all three hyperparameters are set to 0.001, i.e., $\lambda_{1}=\lambda_{2}=\lambda_{3}=0.001$, to ensure that each branch maintains relatively balanced optimization strength during joint training.

## 4. Experiments

This section aims to systematically evaluate the performance of the proposed CD-TriMamba model. Based on the constructed dataset and evaluation metrics, the model’s effectiveness is validated through quantitative experiments and visualized results, and its computational complexity and classification performance are compared with mainstream SOTA methods. Finally, ablation studies are conducted to analyze the individual contributions of each module, revealing the key factors behind the performance improvement of the CD-TriMamba model. The CD-TriMamba network ran on Ubuntu 20.04, with an NVIDIA RTX 3060 GPU (10 GB VRAM, NVIDIA Corporation, Santa Clara, CA, USA), Python 3.8, PyTorch 2.0.1, and CUDA 11.7 as the runtime environment.

### 4.1. Datasets

In this study, we utilized the open-source maize kernel dataset provided by GaoZhe Tech(Anhui Gaozhe Information Technology Co., Ltd., Hefei, Anhui, China), along with our self-collected SS-M-Dataset. The GaoZhe training set contains a total of 11,460 samples, encompassing two major categories: normal kernels (NOR, 4800 samples) and defective kernels (DU, 6660 samples). The defective category is further subdivided into six subclasses: wilted (FM, 1344 samples), sprouted (SD, 804 samples), moldy (MY, 1344 samples), broken (BN, 1344 samples), pest-damaged (AY, 1344 samples), and black-spotted (BP, 480 samples). This hierarchical categorization provides a reliable data foundation for modeling and evaluating maize kernel classification tasks. To clearly reveal the composition characteristics of the dataset, we conducted a systematic statistical and quantitative analysis of its class distribution, laying the groundwork for subsequent experimental design and model performance evaluation.

In contrast, our team independently constructed a comprehensive corn kernel dataset named SS-M-Dataset, which contains both visible-light and hyperspectral images. The dataset demonstrates significant advantages in terms of scale and distribution. Specifically, the visible-light subset contains a total of 9400 samples, including 4000 normal kernels and 5400 defective kernels, comprehensively covering multiple typical imperfect categories such as shriveled, germinated, moldy, cracked, insect-damaged, and black-spotted kernels. The class distribution of the visible light subset is presented in Table 1. Regarding imaging conditions and data acquisition, the visible-light dataset includes diverse images collected under varying illumination, viewing angles, and background conditions. The hyperspectral data were acquired using the GaiaSorter-RT17 push-broom hyperspectral imaging system. During acquisition, the built-in halogen lamps provided stable and uniform broadband illumination, while the exposure time and conveyor speed were carefully matched to ensure imaging consistency. The kernel samples were evenly spread on the conveyor belt and moved at a constant speed, while the vertically mounted line-scan camera dynamically captured the reflected spectral signals of the kernels line by line through the camera slit. Subsequently, the continuously scanned two-dimensional spatial images were synchronously combined with one-dimensional spectral reflectance information to generate three-dimensional hyperspectral data cubes containing rich internal and external spectral–spatial characteristics of the corn kernels.

A total of 20,860 maize kernel samples were used in this study for model training and validation. To systematically evaluate the classification performance under different data split conditions, three training–validation split schemes were designed: 7:3, 8:2, and 9:1. The results indicate that the split ratio has a noticeable impact on model performance. In the 7:3 split, the validation set is relatively large, providing a good reflection of the model’s generalization ability, but the limited training samples may lead to underfitting in complex class recognition. In the 9:1 split, the training set is maximized, enhancing the model’s learning capability, but the small validation set size results in high variance and reduced evaluation stability. The 8:2 split offers a reasonable balance between training and validation sets, allowing sufficient feature learning while achieving robust evaluation on an adequately sized validation set. Therefore, the 8:2 training–validation split is adopted in subsequent experiments to ensure reliable and representative model performance assessment.

### 4.2. Data Standards

We constructed a high-quality maize kernel image dataset covering diverse real-world scenarios, comprising tens of thousands of images and thousands of unique kernel samples. The dataset includes maize varieties from different regions across China to ensure diversity and representativeness. Image acquisition was primarily conducted indoors, while covering various lighting conditions such as natural light, incandescent lamps, and LEDs, including extreme scenarios like strong backlighting and occlusions, to enhance model generalization. The acquisition devices included high-definition cameras, mobile devices, and microscopic imaging equipment, ensuring high resolution and rich spatial information. Professionally, all kernels were manually sorted and annotated according to health status and abnormal types, including normal kernels and abnormal kernels (e.g., insect damage, disease spots, breakage, germination, mold, and heat damage). Each image retains complete environmental context information, such as background setup and shooting angles, to improve the practical applicability and robustness of the model in real detection and classification scenarios.

To achieve fine-grained recognition and standardized evaluation, we designed a maize kernel classification assessment scale (Table 2), tailored to the requirements of image recognition tasks. The scale covers classification dimensions, evaluation criteria, descriptive information, and improvement suggestions. Based on common kernel conditions observed in production and inspection, abnormal kernels are further divided into multiple subclasses, enabling the model to accurately identify different defect types and perform efficient classification and quality assessment. This assessment scale is grounded in the national maize standard (GB 1353-2018 [31]) and seed quality management regulations (GB/T 3543.1-2025 [32]), incorporating key indicators such as kernel morphology, color, surface defects, and integrity. The scale provides clear annotation guidelines for classification models and lays a foundation for intelligent quality inspection and grading, facilitating automated recognition, precise feedback, and smart management.

### 4.3. Evaluation Metric

After completing model training, the performance of TriMamba was systematically evaluated. To comprehensively assess the hand gesture recognition system on the test set, multiple key metrics were analyzed, including Accuracy (Acc), Precision (Pre), Recall (Re), mean Average Precision (mAP), and the Kappa coefficient. The mathematical definitions of these metrics are as follows:

(29) $$\mathrm{Acc}=\frac{\mathrm{TP}+\mathrm{TN}}{\mathrm{TP}+\mathrm{TN}+\mathrm{FP}+\mathrm{FN}}$$

(30) $$\mathrm{Pre}=\frac{\mathrm{TP}}{\mathrm{TP}+\mathrm{FP}}$$

(31) Re=TPTP+FN

Here, TP (True Positive) denotes pixels where both the ground truth and the prediction correspond to the grain region, while TN (True Negative) denotes pixels where both correspond to the background. FP (False Positive) and FN (False Negative) correspond to pixels where the ground truth and prediction disagree.

(32) $$\mathrm{mAP}=\frac{1}{N}\sum_{i=1}^{N}\mathrm{AP}$$

(33) AP=∫01Pre(Re)dRe

Additionally, the Kappa coefficient is used to assess consistency and can also measure classification accuracy. It is calculated as follows:

(34) $$\mathrm{Kappa}=\frac{p_{o}-p_{e}}{1-p_{e}}$$

The Kappa coefficient ranges from [−1, 1]. A value of k=1 indicates perfect agreement; k=0 indicates agreement equivalent to random classification; and k<0 indicates worse-than-random agreement. Here, $p_{o}$ denotes the observed agreement (i.e., overall accuracy, OA), calculated as the ratio of correctly classified samples to the total number of samples. $p_{e}$ represents the expected probability of random agreement, computed as follows:

(35) $$p_{e}=\frac{\sum_{i=1}^{m}A_{i}\times B_{i}}{N\times N}$$

Here, m denotes the total number of classes, N  denotes the total number of samples, $A_{i}$ represents the number of true samples in class i, and $B_{i}$ represents the number of samples predicted as class i. Generally, the higher the Kappa coefficient of a model, the stronger the agreement between its classification results and the ground truth, and the better its robustness.

### 4.4. Experimental Details

**Figure 7:**
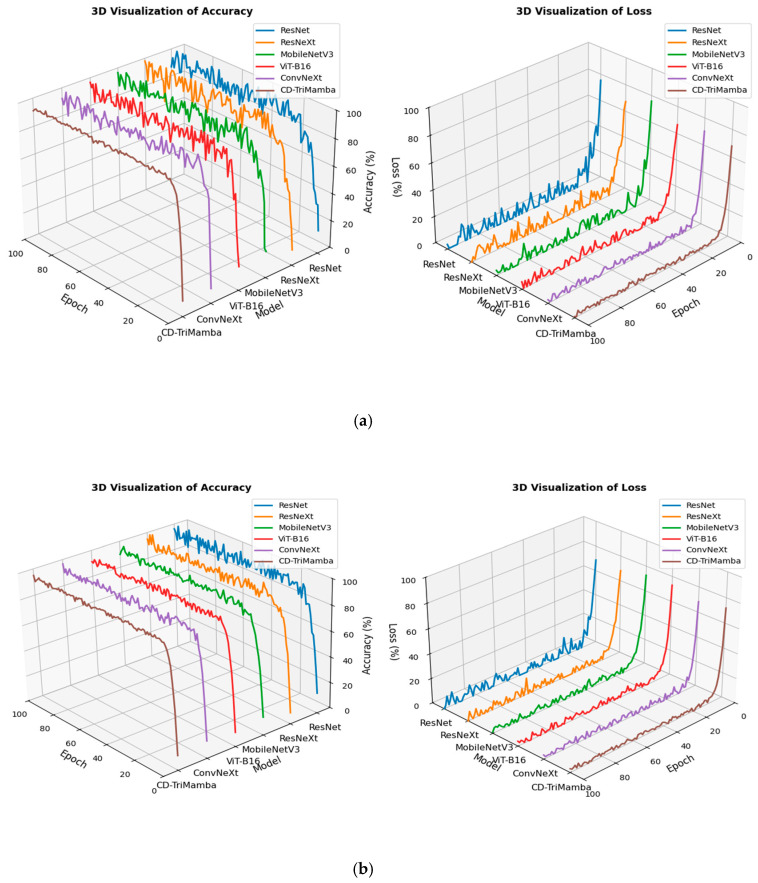
Training accuracy and loss curves of different models on two datasets: (**a**) GaoZhe dataset; (**b**) SS-M dataset.

To evaluate the performance of the TriMamba model, this study conducted comparative analyses from three perspectives:

(1) Performance on the SS-M-Dataset: TriMamba was compared with various classical and state-of-the-art models to demonstrate its advantages and applicability under identical data conditions.
(2) Cross-dataset training accuracy assessment: The training performance of TriMamba on different datasets was examined to verify its stability and generalization capability across diverse data conditions.
(3) Comparison with mainstream models on the same task: Comparative experiments with current mainstream methods were conducted to further validate the effectiveness and superiority of TriMamba in hand posture recognition tasks. Figure 7 illustrates the training process of all models. The accuracy curve of CD-TriMamba exhibits a clear and continuous upward trend, stabilizing around the 40th epoch. Meanwhile, the loss curve decreases rapidly with the increase in training iterations, eventually maintaining a low level. This indicates that CD-TriMamba demonstrates remarkable stability and convergence speed during the training process, highlighting its distinct advantages. To further validate the effectiveness of the constructed SS-M dataset, five representative recognition methods were selected for comparison. Compared to the GaoZhe dataset, the accuracy curve on the SS-M dataset exhibits less fluctuation, faster convergence, and the loss curve also converges more quickly. As shown in Figure 7a, the performance metrics on the SS-M dataset consistently outperform those on the GaoZhe dataset across all five methods, demonstrating the significant advantages of the SS-M dataset in model training and performance evaluation. It is particularly noteworthy that the ConvNeXt method shows exceptional performance on the SS-M dataset, further emphasizing the potential of this dataset in enhancing the performance of advanced models. The outstanding performance of the CD-TriMamba method, especially its superior performance on the SS-M dataset, confirms its value as an efficient training approach with significant practical application potential.

**Figure 8:**
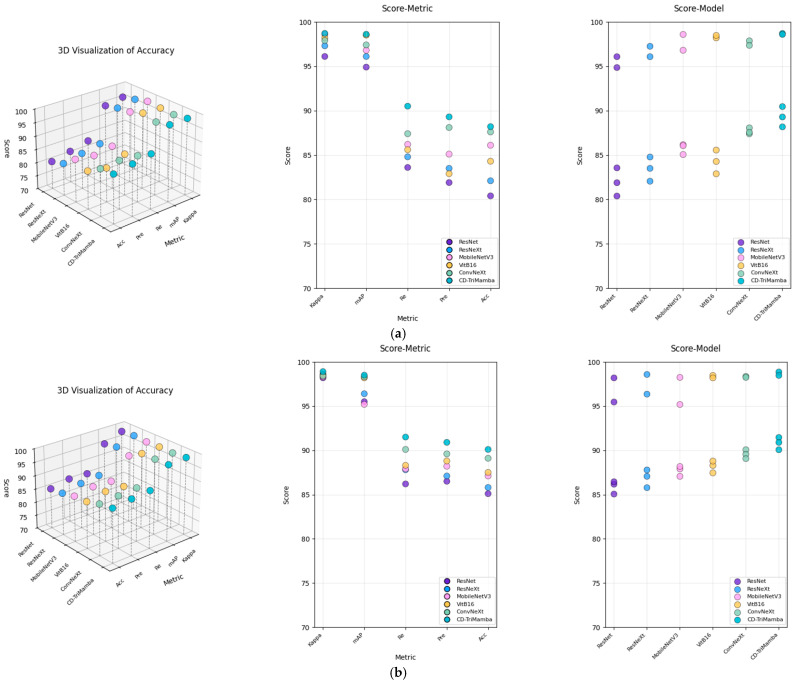
Performance analysis using five evaluation metrics for methods across various datasets: (**a**) GaoZhe dataset; (**b**) SS-M dataset.

To further validate the effectiveness of the constructed SS-M-Dataset, we conducted a comprehensive comparison of five representative classification methods, with the results presented in Figure 8. We performed quantitative evaluations on both the GaoZhe dataset and the SS-M Dataset. While both datasets exhibit high overall performance, the SS-M-Dataset demonstrates a more stable and concentrated data distribution across multiple metrics. Figure 8a shows the experimental results on the GaoZhe dataset, where the performance on 3D images displays considerable fluctuations. In contrast, the SS-M-Dataset exhibits a more concentrated and stable data distribution, as shown in Figure 8b. Similarly, the 2D projections of both datasets follow the same distribution trend. A further analysis reveals that the proposed TriMamba model exhibits significant performance advantages across several key metrics, including accuracy (Acc), precision (Pre), recall (Re), mean average precision (mAP), and Kappa coefficient. TriMamba demonstrates outstanding performance on both datasets, with the Kappa coefficient and mAP exceeding 95%, and the other three metrics being no lower than 75%, indicating the overall high performance of both datasets.

Additionally, on the GaoZhe dataset, TriMamba achieved a notable improvement in overall accuracy compared to ResNet, ResNeXt, and ConvNeXt, with increases of approximately 3.9%, 2.4%, and 1.1%, respectively. On the SS-M-Dataset, these performance improvements were further enhanced, with accuracy increasing by 4.8%, 3.7%, and 2.1%, respectively. This result demonstrates that the TriMamba model maintains a high level of consistency across different data distributions, with performance on SS-M-Dataset consistently exceeding 90% on all metrics, further highlighting the robustness of the model.

#### 4.4.1. Spectral Signal Analysis

To systematically evaluate the robustness of the TriMamba model under different signal-to-noise ratio (SNR) conditions, Gaussian noise at levels of 10 dB, 20 dB, 30 dB, 40 dB, and 50 dB was added to the original spectral data, and the results were compared with the noise-free baseline. During training, noisy spectra were used as inputs, while the fine-tuning stage employed only noise-free spectra, allowing assessment of the transfer effect of pretraining under varying noise conditions.

As shown in Table 3, with a moderate increase in noise (i.e., SNR rising within a certain range), TriMamba’s performance exhibits a trend of first increasing and then decreasing. The optimal performance is observed at SNR = 40 dB, where overall accuracy (Acc) and the Kappa coefficient reach 99.2% and 99.6%, respectively. This indicates that an appropriate amount of noise can enhance the model’s robustness to spectral features, enabling it to learn more stable and discriminative representations. However, when the SNR is further reduced (e.g., 10 dB and 20 dB), excessive noise interferes with feature extraction, resulting in performance degradation, even below that of the baseline encoder (BE) without added noise. This demonstrates that overly strong noise can weaken the model’s discriminative ability.

Further comparisons indicate that the Denoising Encoder (DE) can effectively suppress interference and extract robust features under moderate noise levels. The pretrained DE consistently outperforms the non-pretrained baseline across different SNR conditions. Specifically, compared with the non-pretrained model, the average accuracy (Acc) of the DE-pretrained model improves by 0.6–5.7%, and the Kappa coefficient increases by 0.9–3.4%. This demonstrates that pretraining on noisy spectra enables the model to extract more generalizable knowledge from the raw signals, facilitating effective transfer and performance enhancement in downstream tasks.

#### 4.4.2. Network Performance Analysis

In terms of model comparison, although Mamba already outperforms the non-pretrained traditional CNN in feature extraction, the TriMamba pretrained model guided by the ConvNeXt structure achieves remarkable improvements in both accuracy (Acc) and Kappa coefficient, demonstrating stronger discriminative power and generalization capability. These results fully validate the proposed architecture’s comprehensive advantages in both performance and efficiency. Notably, when TriMamba reaches an Acc of 99.2%, mAP of 99.6%, and Kappa of 99.1%, it maintains extremely high precision while significantly reducing computational overhead, highlighting its excellent adaptability and potential for practical application in maize kernel classification tasks.

When the SNR of noisy spectral data reaches 40 dB, the denoising encoder achieves optimal gains, significantly enhancing downstream maize kernel classification performance. Based on this, the study further analyzed the impact of different datasets on the performance and computational complexity of the CD-TriMamba model. As shown in Table 4, we compared the performance of CD-TriMamba on the GaoZhe-Dataset and SS-M-Dataset with and without Mamba pretraining, alongside the FLOPs and parameter scales (Params) of three representative CNN methods. Results indicate that without Mamba pretraining, CD-TriMamba achieves FLOPs of 0.62 M on GaoZhe-Dataset and 0.65 M on SS-M-Dataset, representing reductions of 77.69% and 77.16% compared to the average FLOPs of CNN-based methods, demonstrating substantial computational efficiency. Moreover, its parameter count significantly decreases, with reductions of 92.85% and 93.20% relative to CNN methods, further validating the model’s lightweight design. Overall, the results show that CD-TriMamba maintains high classification accuracy across different datasets while substantially reducing computational complexity and model size, highlighting its efficiency, scalability, and suitability for online maize kernel classification tasks.

**Figure 9:**
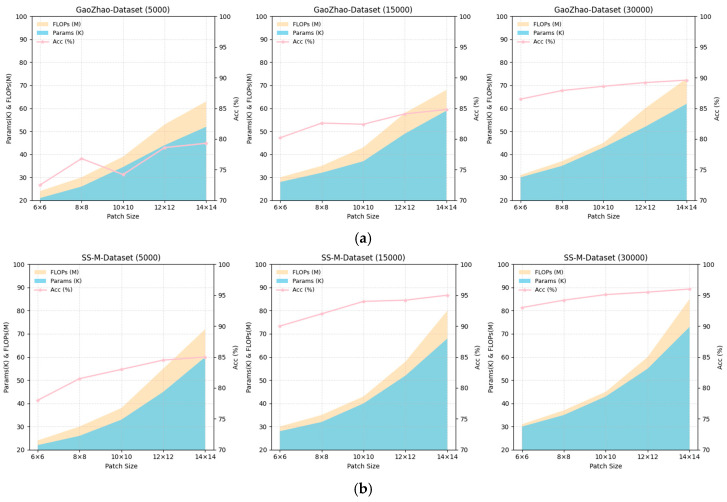
Acc, Params, and FLOPs using different patch sizes on two datasets: (**a**) GaoZhe dataset; (**b**) SS-M dataset.

To more intuitively validate the superiority of the CD-TriMamba method, Figure 9 compares the performance of the proposed model across two datasets with different data volumes. Overall, as the data volume increases, the model’s recognition accuracy gradually improves, and both the parameter count and FLOPs stabilize. However, on the GaoZhe dataset, when the patch size is 10 × 10, the model exhibits larger fluctuations. In contrast, the SS-M dataset shows a more stable improvement trend. Moreover, batch size has a significant impact on model performance. At a 30,000 data volume, when the batch size increases from 6 × 6 to 8 × 8, the classification accuracy significantly improves: on the GaoZhe dataset, accuracy increases from 87.43% to 88.2%, while on the SS-M dataset, accuracy rises from 94.68% to 96.47%. More importantly, this improvement does not significantly increase the computational load, as the model remains lightweight (Params ≈ 71.9 KB, FLOPs ≈ 0.85 MB). These results demonstrate that the CD-TriMamba method strikes an ideal balance between performance and computational efficiency, offering strong classification capabilities while remaining highly efficient for real-time corn kernel classification tasks.

To further validate the reliability and robustness of the proposed method, repeated experiments were conducted under identical experimental settings, and the mean performance together with standard deviation values were reported, as shown in Table 5. The statistical analysis demonstrates that the proposed framework maintains stable and consistent classification performance across different experimental runs.

### 4.5. Ablation Studies

***A. Analysis Across Datasets.***

In this section, we conducted systematic ablation experiments on the SCC and CDC branches within the CD module. The results (Table 6) demonstrate significant performance differences among the four tested configurations. First, when both SCC and CDC are removed, classification accuracy drops substantially, indicating that frequency information in hyperspectral data is indispensable for effective feature representation. Second, retaining only SCC or only CDC can improve performance, with SCC outperforming CDC in accuracy, suggesting that spatial frequency features alone provide stronger discriminative power than spectral frequency features. However, using either branch individually does not achieve optimal results. Ultimately, when SCC and CDC are combined, the model attains the best performance across all datasets. This is because SCC extracts multi-scale spatial frequency features through 2-D curvelet decomposition, while CDC separates the low-frequency trend and high-frequency details of spectral curves via 1-D curvelet decomposition, complementing each other to enable multi-dimensional feature synergistic modeling. Overall, these findings validate the complementarity and synergy of SCC and CDC, further confirming the necessity and effectiveness of the CD module design in hyperspectral image classification tasks.

***B. Analysis Across Branches.***

In this section, we conducted a systematic cross-branch ablation study on the spectral, spatial, and ConvNeXt-guided branches of the CD-TriMamba model to evaluate their individual contributions. First, the spectral branch, based on curvelet decomposition, effectively captures low-frequency trends and high-frequency details in spectral curves, enhancing the model’s ability to discriminate material and compositional differences. However, relying solely on the spectral branch results in reduced overall classification performance due to the lack of spatial structure modeling. Second, the image-based spatial branch excels at extracting local structural information such as textures and edges, showing strong adaptability to geometric features in high-resolution images; yet, without spectral support, it struggles to achieve comprehensive representation in complex scenes, limiting classification accuracy. In contrast, the ConvNeXt-guided branch provides stable convolutional priors and facilitates feature convergence, improving training stability and generalization, but its effectiveness depends on integration with spectral and spatial information.

When all three branches operate collaboratively, the model achieves optimal performance across datasets: the spectral branch contributes material discrimination, the spatial branch strengthens geometric analysis, and the ConvNeXt-guided branch provides stable priors, enabling complementary and enhanced multi-dimensional feature representation. These results demonstrate that only through the fusion of these three branches can the model maintain high accuracy while ensuring efficiency and robustness.

As shown in Table 7, we first conducted a comparative analysis of the performance of each sub-branch within the CD-TriMamba method to validate its effectiveness in maize recognition. Here, “Spectral” denotes the spectral branch, “Spatial” denotes the spatial branch, and “ConvNeXt” denotes the ConvNeXt-guided branch. Using CD-TriMamba as the baseline, the fusion of the “Spectral” and “Spatial” branches increased the recognition accuracy by 1.4% and 1.1%, respectively, compared with using each branch individually. This notable improvement highlights the critical role of the spectral branch in feature extraction and underscores the importance of multi-branch collaborative fusion in enhancing the overall performance of CD-TriMamba.

Furthermore, the CD-TriMamba method integrates all three branches: “Spectral,” “Spatial,” and “ConvNeXt.” Compared to using any single branch, CD-TriMamba achieves the best performance in terms of accuracy, Kappa coefficient, and mAP metrics, with the overall recognition accuracy improving by 4.6% over the “Spatial” branch alone. By further incorporating the ConvNeXt-guided branch, the model’s Kappa coefficient and mAP increase by 1.1% and 0.6%, respectively, further validating the effectiveness of the multi-branch feature fusion strategy.

***C. Ablation Matrix.***

**Figure 10:**
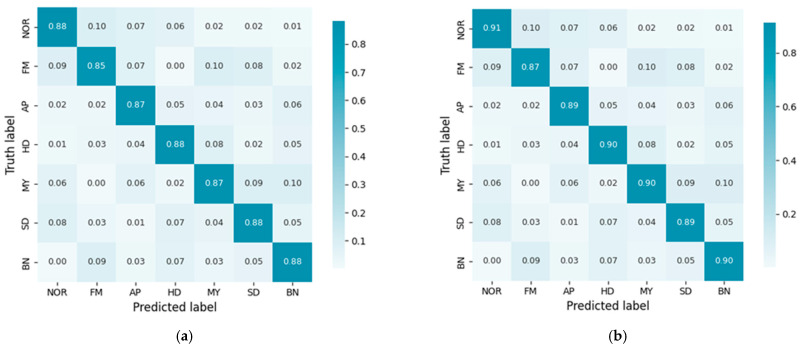
The confusion matrices on the GaoZhe dataset and SS-M dataset: (**a**) GaoZhe dataset; (**b**) SS-M dataset.

To comprehensively evaluate the recognition performance across different classes, Figure 10 presents the confusion matrices of the CD-TriMamba model on the GaoZhe-Dataset and SS-M-Dataset. Here, “NOR,” “FM,” “AP,” “HD,” “MY,” “SD,” and “BN” correspond to seven categories of maize kernels. By comparing the predicted results with the ground truth labels, the classification performance for each category can be visually assessed. Given the high overall accuracy of the CD-TriMamba model, most predictions are concentrated along the main diagonal. As shown in Figure 10, the majority of categories are accurately recognized, with recognition accuracy exceeding 85% across both datasets. Notably, the “NOR” category achieves the best performance on the SS-M-Dataset, with an accuracy of 91.2%. These results clearly demonstrate the superiority of CD-TriMamba in maize kernel recognition, providing stable and efficient classification performance.

### 4.6. Discussion

During spectral data acquisition, environmental noise and equipment errors inevitably affect data quality. Traditional studies often rely on various spectral preprocessing techniques to suppress noise and improve data reliability. However, the choice of preprocessing methods is highly uncertain, with significant performance differences among strategies, and excessive reliance on complex preprocessing may introduce additional errors, undermining model robustness. In contrast, deep learning models, due to their data-driven nature, can operate directly on raw spectral data and automatically extract discriminative features, reducing the dependency on cumbersome preprocessing. Previous studies have shown that deep models trained on raw spectra can achieve comparable or even superior performance to traditional preprocessing-based methods across multiple tasks.

In this context, research combining spectral and image modalities has gained attention. Existing works have demonstrated the effectiveness of spectral–image fusion in food inspection, medical diagnosis, and material identification, yet its application in seed classification and quality assessment remains limited. Seed images primarily provide surface morphology and texture features, while hyperspectral data reflect the internal chemical composition and spectral response of kernels, exhibiting natural complementarity. Fully leveraging this complementarity for deep cross-modal fusion is crucial for improving classification accuracy and robustness.

Based on this rationale, we propose the CD-TriMamba cross-modal classification framework, designed to exploit the advantages of both hyperspectral and visible-light images. Specifically, hyperspectral data are processed through the SCC and CDC modules to capture multi-scale, multi-directional frequency and spectral–spatial features, while the image branch focuses on surface texture and structural information. Through a three-branch fusion mechanism, the model achieves deep interaction between modalities, yielding more robust and discriminative feature representations. Experimental results demonstrate that this framework excels in imperfect seed recognition tasks, achieving significant improvements in classification accuracy, generalization, and stability compared to single-modality approaches. This finding not only validates the complementarity of hyperspectral and image modalities but also highlights the potential of cross-modal fusion in rapid, non-destructive maize seed inspection.

Furthermore, the superior ACC and Kappa results indicate that the proposed framework can effectively reduce inter-class confusion and improve classification consistency under complex imaging conditions. Compared with conventional CNN-based and single-modal methods, the proposed framework demonstrates stronger robustness in distinguishing subtle defect categories, which can be attributed to the collaborative learning of spectral–spatial frequency features and visible structural information. Similar findings have also been reported in previous studies on multimodal hyperspectral fusion and state-space sequence modeling, confirming the effectiveness of combining complementary modalities for agricultural inspection tasks. Nevertheless, several limitations still exist in the current framework. The integration of cross-modal fusion and dual-domain dependency modeling increases computational complexity and memory consumption, which may restrict deployment on lightweight edge devices. In addition, the proposed method still relies on high-quality hyperspectral data acquisition and may exhibit sensitivity to severe data distribution shifts or unseen defect categories in practical industrial environments. Therefore, future work will focus on lightweight model optimization, cross-device generalization, and real-time industrial deployment.

## 5. Conclusions

This study addresses the challenges of strong spectral feature dependence, complex pre-processing, and insufficient multimodal fusion in rapid, non-destructive maize kernel classification by proposing a cross-modal deep learning framework, CD-TriMamba. The framework leverages Spectral Curvelet Convolution (SCC) and Decoupled Convolution (CDC) modules to effectively enhance the spectral–spatial representation of hyperspectral data across multiple scales and orientations, while integrating texture and morphological features from visible images to achieve deep cross-modal feature fusion. Different from existing CNN-based methods that mainly rely on local feature extraction and Transformer-based approaches that emphasize global attention but often incur high computational cost, the proposed method is positioned as a unified spectral–spatial and cross-modal learning framework that jointly models multi-scale local structures and long-range dependencies. This positioning bridges the gap between local representation learning and global dependency modeling in existing studies, providing a more balanced and efficient solution for imperfect maize kernel classification.

Experimental results demonstrate that the pre-trained CD-TriMamba model achieves outstanding performance in maize kernel classification, with an overall accuracy (Acc) of 99.2% and a Kappa coefficient of 99.1%, significantly outperforming the non-pretrained model (Acc = 93.5%, Kappa = 95.7%). In terms of classification accuracy, generalization capability, and computational efficiency, CD-TriMamba surpasses existing state-of-the-art methods, confirming the effectiveness of cross-modal fusion in maize kernel classification and quality assessment. Compared with traditional methods that rely on spectral preprocessing, this approach eliminates labor-intensive parameter tuning, realizing end-to-end deep learning modeling and highlighting its practical potential.

In summary, the proposed CD-TriMamba framework effectively integrates hyperspectral and visible-light information through spectral–spatial collaborative modeling and cross-modal feature fusion, achieving superior classification accuracy, robustness, and generalization performance in imperfect maize seed recognition. The proposed method demonstrates strong practical potential for rapid and non-destructive agricultural inspection applications. In future work, we will further evaluate the framework on other crop seeds and agricultural products, while exploring lightweight optimization and cross-domain adaptation strategies to improve industrial deployment capability and model generalization.

## Figures and Tables

**Table 1:** Dataset distribution.

Classes		GaoZhe Dataset			SS-M-Dataset		
		Train	Validation	Test	Train	Validation	Test
DU	FM	1344	480	480	1000	500	500
	SD	804	216	216	800	400	400
	MY	1344	480	480	1000	500	500
	BN	1344	480	480	1000	500	500
	AY	1344	480	480	1000	500	500
	BP	480	120	120	600	300	300
NOR		4800	2400	2400	4000	2000	2000

**Table 2:** Maize seed evaluation form.

Class	Seeds	Evaluation	Description	Suggestion
NORMAL (NOR)	Normal maize kernels	OK	Normal grains.	Null
FUSARIUM (FM)	Moldy maize kernels	NG	Particles that have mold growing on their surface.	Strict detection and removal to ensure safety.
ATTACKED PESTS (AP)	damaged maize kernels	NG	Grains that have been eaten by insects and formed wormholes or tunnels.	Strengthen insect inspection and remove affected seeds.
HEATED (HD)	Heated maize kernels	NG	Particles that have undergone significant color change or damage due to heat, either during heating or after being exposed to heat, including natural heat-damaged particles and dried heat-damaged particles.	Implement temperature monitoring and remove affected seeds.
MOULDY (MY)	Mouldy maize kernels	NG	Grains with lesions on the surface that affect the embryo or endosperm.	Prioritize identification and isolation to prevent contamination.
SPROUTED (SD)	Sprouted maize kernels	NG	Young buds or young roots break through the epidermis, or although the buds or roots have not broken through the epidermis, the epidermis of the embryo part has ruptured or is significantly bulging, with signs of budding present in the particles.	Clearly classify as defective seeds.
BROKEN (BN)	Broken maize kernels	NG	Grains with fragmentation reaching one fifth (inclusive) or more of the volume of the original particle.	Classify and remove to avoid mixing with healthy seeds.

**Table 3:** Performance of pre-trained models under noisy spectra with different signal-to-noise ratios (SNRs).

Models	SNR (dB)	Acc (%)	Kappa (%)	mAP (%)
Pre-train Mamba	10	95.6	96.3	98.4
	20	97.2	97.5	98.7
	30	98.4	98.2	98.9
	40	99.2	99.1	99.6
	50	98.6	98.4	99.2
	No noise	97.2	97.5	98.1
Non-Pre-train Mamba	/	93.5	95.7	97.6

**Table 4:** Number of parameters and FLOPs for DL models.

Models	GaoZhe Dataset		SS-M Dataset	
	FLOPs (MB)	Params (MB)	FLOPs (MB)	Params (KB)
Non-Pre-train Mamba	0.62	13.58	0.65	11.55
Pre-train Mamba	0.71	13.67	0.76	12.87
ResNet-18 [33]	2.78	14.3	2.72	13.92
ResNet-152 [33]	446.5	58.2	442.2	57.2
ResNeXt-50 [34]	182.19	24	181.9	25.1
ResNeXt-101 [34]	696.8	87	695.1	85.2
MobileNetV3 small [35]	12.8	1.8	12.5	1.75
MobileNetV3 large [35]	34	4.3	33.2	4.8
VitB16 [16]	687	85.4	675	83.4
ConvNeXt V1 [36]	173	88.6	151	80.1
ConvNeXt V2 [37]	176	89.1	156	82.4
RetinaNet [38]	295	38.2	294	37.5
SSDLiteMobileNetV3 small [39]	9.4	1.9	9.1	2.3
SSDLiteMobileNetV3 large [39]	18.8	3.5	17.6	3.3

**Table 5:** Statistical results from multiple experiments on the GaoZhe Dataset and SS-M-Dataset.

Datasets	Count	Acc (%)	Kappa (%)	mAP (%)
GaoZhe Dataset	1	97.6	97.7	98.4
	2	97.2	97.5	98.7
	3	97.4	97.4	98.5
	4	97.2	97.1	98.6
	5	97.6	97.4	98.2
	6	97.2	97.5	98.1
Mean(%)		97.36	97.43	98.42
SS-M Dataset	1	97.9	98.3	99.4
	2	98.1	98.5	99.3
	3	98.2	98.4	99.5
	4	99.2	98.2	99.3
	5	98.1	98.4	99.2
	6	98.2	98.5	99.1
Mean(%)		98.12	98.38	99.3

**Table 6:** Acc with and without SCC and CDC modules across different datasets.

CD Block		Acc (%)	Pre (%)	Kappa (%)	mAP (%)
SCC	CDC				
×	×	89.1	89.1	97.8	98.1
√	×	86.3	85.9	98.5	98.3
×	√	89.6	91.3	98.1	98.2
√	√	91.2	91.3	98.6	98.7

**Table 7:** Acc with and without SCC and CDC modules across different datasets.

Branch			Acc (%)	Pre (%)	Kappa (%)	mAP (%)
Spatial	ConvNext	Spectral				
×	√	×	89.1	89.1	97.8	98.1
×	√	√	86.3	85.9	98.5	98.3
√	×	×	86.6	91.1	98.1	98.2
√	×	√	87.7	87.4	97.5	98.1
√	√	×	89.2	90.8	98.2	98.5
√	√	√	91.2	91.3	98.6	98.7

## Data Availability

The raw data supporting the conclusions of this article will be made available by the authors on request.
